# Optimized methods for random and targeted mutagenesis in field pea (*Pisum sativum* L.)

**DOI:** 10.3389/fpls.2022.995542

**Published:** 2022-09-08

**Authors:** Prashant Kumar Pandey, Pankaj Bhowmik, Sateesh Kagale

**Affiliations:** Aquatic and Crop Resource Development, National Research Council Canada, Saskatoon, SK, Canada

**Keywords:** gamma radiation, ethyl methanesulfonate, protoplasts, CRISPR/Cas (clustered regularly interspaced short palindromic repeats), legume crops, artificial mutagenesis

## Abstract

Field pea is an important pulse crop for its dense nutritional profile and contribution to sustainable agricultural practices. Recently, it has received extensive attention as a potential leading source of plant-based proteins. However, the adoption of peas as a mainstream source of proteins is affected by a relatively moderate protein content, anti-nutritional factors and high levels of off-flavor components that reduce protein quality. Availability of genetic variation for desirable seed quality traits is the foundation for the sustainable development of pea varieties with improved protein content and quality. Mutagenesis has been an important tool in gene functional characterization studies and creating genetic variability for crop breeding. Large-scale mutagenesis of a crop using physical and chemical agents requires diligent selection of the mutagen and optimization of its dose to increase the frequency of mutations. In this study, we present detailed optimized protocols for physical and chemical mutagenesis of pea using gamma irradiation and ethyl methanesulfonate (EMS), respectively. Gamma radiation and EMS titration kill curves were established to identify optimal doses of the two mutagenic agents. Based on germination, survival rate and growth phenotypes, a gamma radiation dose of 225 Gy and EMS concentration of 5 mm were selected as optimal dosages for mutagenesis in field pea. The presented protocol has been modified from previously established mutagenesis protocols in other crop plants. Our results indicate that the optimal mutagen dosage is genotype dependent. CRISPR/Cas-based gene editing provides a precise and rapid method for targeted genetic manipulation in plants. With the recent success of gene editing in pea using CRISPR/Cas, this innovative technology is expected to become an integral component of the gene discovery and crop improvement toolkit in pea. Here, we describe an optimized methods for targeted mutagenesis of pea protoplasts, including mesophyll protoplast extraction, PEG-mediated transformation and gene editing of a LOX gene using CRISPR/Cas system. The general strategies and methods of mutagenesis described here provide an essential resource for mutation breeding and functional genomics studies in pea. These methods also provide a foundation for similar studies in other crops.

## Introduction

Field peas, also known as dry peas, are a member of the third largest family of flowering plants Fabaceae (formerely Leguminosae). They are a rich source of prebiotic carbohydrates, protein, and micronutrients, such as Iron and Zinc. Pea seeds hold approximately 70% of the above-ground biomass protein (Ranjbar Sistani et al., 2017), which is in higher concentration and of superior quality than chickpea and cowpea (Iqbal et al., 2006). Due to its affordability, the field pea is judicially hailed as an important crop for eradicating hidden hunger in the world. The protein-rich seed profile also makes pea an important crop for the exponentially growing plant-based protein and animal-feed industry (Sim et al., 2021). Field peas are also favored in crop rotation and sustainable agricultural practices as they enrich the soil with organic matter and nitrogen, and reduce weed, disease, and pest pressures on the subsequent cereal crop (Stagnari et al., 2017). Field pea cultivation, however, faces certain challenges that must be addressed to realize its full commercialization potential.

Pea is a cool-season crop and highly sensitive to heat stress, which is predicted to increase in frequency and intensity in the future due to climate change. Similarly, the rapid spread of pea beyond its natural ecological niche has made it vulnerable to diseases and pests (Rawal and Navarro, 2019). Agronomically, there is a demand to enhance pea yields, and improve protein and starch content in the seed for commercial and nutritional purposes. There is also considerable but, thus far, unrealized potential of biofortification of mineral nutrients in the crop, which will drastically improve its nutritional profile.

Presence of genetic variation is essential for development of new varieties with improved agronomic and seed quality traits to meet the present and future challenges in pea cultivation. Breeders take advantage of natural genetic variation that is generated by spontaneous mutations. Several natural pea germplasm collections have been established, including the USDA and European pea germplasm collections (Jing et al., 2012; Al Bari et al., 2021). The analysis of genetic polymorphisms in these diverse germplasm pools is currently underway (Burstin et al., 2015; Pandey et al., 2021; Shirasawa et al., 2021; Crosta et al., 2022) to facilitate the association of key phenotypes with the identified genetic polymorphisms. However, continuous selection of only yield-related traits among crosses of genetically related pea cultivars has led to narrowing of the genetic base of the crop, particularly affecting the historically neglected protein-related traits. Therefore, complementing traditional breeding approaches with well-functioning supplementary tools, such as mutation breeding and genome editing, is required to create and exploit genetic variation in agronomic and seed quality traits, thereby removing the barriers to the adoption of pea as a leading source of plant-based proteins.

The introduction of novel genetic variation through artificial mutagenesis has accelerated the development of new varieties, functional characterization of genes and the establishment of gene-trait relationships in several crop species (Sikora et al., 2012; Zhu et al., 2012). Random mutagenesis can be carried out using chemicals [such as N-methyl-N-nitrosourea (MNU), sodium azide, ethyl methanesulfonate (EMS)] or physical mutagen agents (gamma rays, X-rays, UV-rays, and fast-moving neutrons), and have been widely used for introduction of genetic variation in model and crop species. These techniques are especially important for the introduction of genetic variation in crops recalcitrant to Agrobacterium-mediated genetic modifications, such as peas (Dalmais et al., 2008). Over the last six decades, 3,402 mutagenized varieties from 257 economically important species have been registered with United Nations International Atomic Energy Agency and FAO.^1^

Mutagenesis can cause a range of genetic variations, including single nucleotide polymorphisms SNPs; leading to either transition (A↔T/G↔C), transversion (A↔G/T↔C), nucleotide insertion or deletion events (indels), chromosomal breaks, and/or chromosomal re-arrangements. Chromosomal breaks or rearrangements can affect multiple genes simultaneously, and are likely to induce the most drastic phenotypes. Smaller effects, such as SNPs and short indels could produce synonymous (non-consequential) or non-synonymous (consequential) effects, with the latter possibly leading to alteration of regulatory regions, premature termination of a gene (nonsense mutation), or alteration of the reading frame (frameshift mutation). The high-energy radiations in physical mutagenesis can cause single-strand nicks or double-strand breaks in the genetic material, predominantly leading to large chromosomal breaks and chromosomal rearrangements, along with SNPs. Chemical mutagenesis, on the other hand, generally causes chemical alterations in specific nucleotides that cellular proof-reading machinery misreads and modifies causing SNPs. For example, EMS causes base changes of G→ A and C→ T.

Induced mutagenesis has found major application in crop improvement programs (mutation breeding), where mutated elite/commercial crop varieties could directly be screened for traits of interest. Unlike genetic modification using transgenic approaches, the mutation breeding approach is accepted by major crop regulatory authorities worldwide. Artificial mutagenesis has also revolutionized basic research by facilitating functional characterization of genes and alleles. Large-scale genotyping and sequencing of such mutagenized populations has led to the establishment of public repositories of genetic variants for several plant species (Dalmais et al., 2008; Chen et al., 2012; Schreiber et al., 2019) which can be used for gene discovery and functional genomics studies (Clemente et al., 2015). Mutagenesis approaches have also been used in generating the much-needed diversity in vegetatively or asexually propagated crops, such as bananas, roots and tubers, ornamental plants (Hernández-Muñoz et al., 2019; Wang et al., 2021), and crops recalcitrant to Agrobacterium-mediated genetic modifications (Dalmais et al., 2008).

While artificial mutagenesis has been tremendously useful in producing improved cultivars and new knowledge of gene functions (Sikora et al., 2012; Nasti and Voytas, 2021), there are technical and practical constraints associated with its application. For example, random mutagenesis is likely to show limited success with polygenic traits, especially in complex polyploid genomes. In such cases, only those alleles which have the highest contribution to a trait are likely to be identified, while the effect of other contributing alleles could be diluted. Also in the case of polyploid crops, the non-mutated homoeologs might compensate the function of the mutated gene/allele, thus masking the phenotypic effect. The random mutagenesis techniques might also show limited success toward epigenetically-regulated phenotypes. This is especially true for chemical mutagenesis where SNPs are less likely to drastically affect the epigenome landscape. However, deletion of an epigenetically modified region using physical mutagenesis is more likely to be useful for such traits. Homozygous mutations often provide clearer read-outs as compared to heterozygous mutations. The fixing of a mutation in subsequent generations of self-pollinating crops, such as pea, makes them easier to work with as compared to the cross-pollinating species. Another practical constraint with the technique is the cost associated with the identification of causal mutations through sequencing techniques.

Artificial mutagenesis has been successfully performed on several plant parts. Mature seeds are the most convenient and commonly used material for mutagenesis as they can be generated in large amounts, and are easy to handle and store. However, other plant materials, such as plant calli, immature inflorescence, isolated immature embryos, anthers, pollen grains, and vegetative propagules have also been used (Joseph et al., 2004; Yang et al., 2004; Ookawa et al., 2014; Serrat et al., 2014; Perera et al., 2015).

The choice between physical or chemical mutagen depends on the aim of the study, the plant material to be mutagenized, and the availability of infrastructure. If a study aims at identifying genes or key nucleotides associated with a trait of interest, then chemical mutagenesis might be preferable. However, if the study aims to identify a genomic locus associated with the trait of interest, especially in an uncharacterized genomic region, then physical mutagenesis might be preferable. Generally, plant seeds are amenable to both physical and chemical mutagenesis, however, large seeds might benefit from high and uniform penetrability of physical mutagens, such as gamma rays or fast-moving neutrons (Oladosu et al., 2016). The high penetrability characteristic of physical mutagens is also associated with the high reproducibility of mutation density (Zheng et al., 2020). However, physical mutagenesis requires access to specialized infrastructure and highly trained personnel to handle the equipment, while chemical mutagenesis could be conducted in a basic laboratory set-up with some precautions.

The optimal dosage of mutagen needs to be quantified diligently. The dosage depends on the plant material and the aim of the study. For a polygenic trait, where multiple genes contribute to a phenotype, high density of mutations might be desirable to allow mutations in multiple genes affecting the phenotype. On the other hand, high density mutations might hinder isolation of specific mutations causing the phenotype due to the background noise. The mutation density is only partially correlated with the mutagen dosage, as high dosages also increase the likelihood of lethality. Therefore, a pilot study to optimize the dose of mutagens is highly recommended.

Here we provide a step-by-step modified protocol for gamma irradiation and EMS treatment of field pea seeds to determine optimal dosages for mutagenesis. We tested several conditions (dosages and time of exposure) on a small batch of field pea seeds (~200 seeds per condition), followed by an analysis of biological effectiveness of the mutagen by measuring germination rate, survival rate and various other growth phenotypes. This analysis helped in the identification of gamma radiation and EMS dosages that allow 50% germination and survival rate (generally termed as lethal dose 50, LD_50_) of field pea seedlings.

Compared to the imprecise and less efficient random mutagenesis, CRISPR/Cas-based genome editing approaches provide a faster way of targeting individual genes or gene families in crop plants. While CRISPR/Cas system appears to work universally, the efficiency of targeted mutagenesis in pea varies greatly because of its recalcitrant nature, and low frequency of transformation and plant regeneration. Continuous efforts are being made by the research community to improve transformation and gene editing in pea (Bhowmik et al., 2021). The first report of successful generation of gene edited pea was recently published (Li et al., 2022). However, to best of our knowledge, CRISPR/Cas-mediated targeted mutagenesis has not been reported in pea protoplasts. Here, we provide the detailed description of methods and procedures for creating targeted edits in pea protoplasts using the CRISPR/Cas system.

## Materials

### Mutagenesis in pea

#### Plant material and growth conditions

Seeds of the field pea genotype CDC Amarillo (Warkentin et al., 2014) obtained from Galloway Seeds (Alberta, Canada) were used for mutagenesis experiments described in this study. After mutagenesis, dry field pea seeds were carefully sown at 3–5 cm depth using blunt-tipped forceps in water-saturated soil mixture (Sunshine mix # 8, SunGro Horticulture, Canada) in 48 pot seeding trays. Each tray was labeled with the mutagen and dose to which the seeds were subjected. The trays were maintained in a growth chamber at 24°C, 16 h/8 h (day/ night), 60% humidity.

#### Chemicals and reagents

1. Ethyl methanesulfonate (Sigma-Aldrich Catalogue number: M0880). EMS (MW 124.16 g/mol) is supplied in liquid form at density of 1.206 g/ml (at 20°C). The working solutions are prepared in water, as described in Table 1. It is important to thoroughly mix the EMS stock in the working solution.
2. Ultra-pure water (to prepare EMS solution and washing of seeds).
3. Sodium thiosulfate (10% w/v in water).

**Table 1:** Preparation of EMS solutions (200 ml) for mutagenesis.

Concentration of working solution (200 ml) (mm)	EMS stock (μl)
5	81.5
10	163
15	303
20	401
25	508
30	606

#### Personal protective equipment

1. Disposable chemical resistant long-sleeved lab coats or coveralls.
2. 3 M half-face reusable respirators (3 M 7000) with particulate filter P100 (3 M 7093).
3. Chemical resistant/industrial nitrile gloves with an extended cuff.
4. Lab safety goggles.
5. Full face shield.

#### Consumables

1. Mesh bags for seeds.
2. Glass beakers (500 ml for EMS treatment, 2 liters for seed washes).
3. Glass stirrer.
4. Long-handled tongs.
5. Magnetic beads (optional).
6. Magnetic stirrers (optional).
7. Pipettes.
8. Filtered pipette tips.
9. Measuring cylinders.
10. Large weighing boats.

### Gamma irradiation of pea seeds

A Gammacell 220 (SN:236) with a ^60^Co source was used for γ-irradiation. At the time of irradiation, the central absorbed dose rate was calculated to be 1.19 Gy/min, and the calibration was confirmed by alanine dosimetry in 2021.

The dose rate was calculated based on the original cell calibration (5,180 Gy/h) using the half-life of ^60^Co with first order decay:


$$N\left(t\right)=N_{0}{\left(\;\frac{1}{2}\;\right)}^{\frac{t}{t_{1/2}}}$$

where N(t) quantity of ^60^Co remaining, $N_{0}$ is the initial quantity of ^60^Co, t is the time elapsed and $t_{1/2}$ is the half-life of ^60^Co, 5.271 yrs.

### Targeted mutagenesis in pea protoplasts using CRISPR/Cas system

Plant protoplasts provide a unique single cell system for conducting cell-based experiments using different molecular, cellular or genomic tools (Davey et al., 2005). With single cell analysis, it is also easier to detect a low frequency of mutated cells than it is with a pooled population. Although many successful reports of CRISPR/Cas mediated gene editing have now been published, effective delivery of genome editing components and early detection of mutation, two keys aspects to achieving high efficiency gene editing, still remain challenging for most of the pulse crops. Successful protoplast isolation depends on careful selection and optimization of several factors including the age of the leaf tissues, duration of enzyme incubation, enzyme concentration, gentle agitation and nature of the osmoticum (Sinha et al., 2003).

#### Plant material

Donor plant leaves of the CDC Meadow pea variety (a cultivar from the Crop Development Centre, University of Saskatchewan; Warkentin et al., 2007) were used for protoplasts isolation.

#### Chemicals and reagents

##### Cell wall enzyme solution (50 ml)

0.6 M Mannitol 5.46 g.
MES 10 mm 1 ml of a 0.5 M Stock.
  Incubate at 70° for 5 min.

##### 1.5% cellulase R-10 0.75 g

0.75% Macerozyme R-10 0.37 g.
Incubate at 55° for 10 min.
CaCl_2_ (10 mm) 0.5 ml of a 1 M Stock.
BSA (0.1%) 1 ml of a 5% Stock.
Bring up to 50 ml, filter sterilize with syringe filter.

##### W5 solution (500 ml)

MES (2 mm) 2 ml of a 0.5 M Stock.
NaCl (154 mm) 77 ml of a 1 M Stock.
CaCl_2_ (125 mm) 62.5 ml of a 1 M Stock.
KCl (5 mm) 2.5 ml of a 1 M Stock.
Bring up to 500 ml, filter sterilize.

##### MMG solution (50 ml)

MES (4 mm) 0.4 ml of a 0.5 M Stock.
0.4 M Mannitol 3.64 g.
MgCl_2_ (125 mm) 0.75 ml of a 1 M Stock.
Bring up to 50 ml, filter sterilize.

##### Peg solution (10 ml)

40% PEG 4000 4.0 g.
0.2 M Mannitol 0.36 g.
CaCl_2_ (1 M) 1 ml of a 1 M Stock.
Bring up to 10 ml.

##### 0.55 M sucrose (250 ml)

Sucrose 47.07 g.
Bring up to 250 ml. Filter sterilize.

##### 0.6 M manitol (500 ml)

Mannitol 54.65 g.
Bring up to 500 ml. Filter sterilize.

##### 0.5 M MES-KOH (50 ml)

MES 4.88 g.
pH to 5.7 with KOH (uses quite a bit). Bring up to 50 ml.

#### Consumables and equipments

1. Surgical blades.
2. 0.22 μm syringe sterilization filter.
3. 15 ml round bottom centrifuge tube.
4. 40 μm nylon mesh.
5. Autoclave.
6. 35 mm Cell culture dish.
7. Pipettes.
8. Filtered pipette tips.
9. Measuring cylinders.
10. Weighing boats.
11. Lamina flow hood.
12. Microcentrifuge and swing-out rotor centrifuge.
13. Plant DNA purification kit.
14. Plasmid purification kit.
15. Thermal Cycler.
16. NanoDrop^™^ Spectrophotometer.

## Methods

### Pre-treatment conditions for chemical and physical mutagenesis

1. Mutagenesis should be performed on a genetically homogeneous seed material to minimize polymorphism in the starting material.
2. Healthy looking seeds of relatively similar size should be selected to ensure uniformity of mutagenesis.
3. Fume hood should be used for chemical mutagenesis. The place of work should be decluttered to minimize spills and droplet contaminations.

### Chemical mutagenesis

EMS, the most widely used chemical mutagen in plants, was selected for chemical mutagenesis of field pea. To determine the optimal dose of EMS, kill curve was established at different concentrations. In total six different EMS concentrations (5, 10, 15, 20, 25, and 30 mm) with a constant incubation period of 18 h were used. The selection of the range of EMS concentrations was guided by previous work on closely related species (Rawal and Navarro, 2019).

#### Ems treatment procedure

1. Aliquots of 200 healthy-looking seeds each for control and the selected EMS doses were prepared in disposable, pre-labeled nylon mesh bags.
2. For each treatment, pea seed bags were fully immersed in 200 ml of EMS solution for 18 h at room temperature. The volume of EMS solution should be sufficient to allow complete submersion of the seeds. The seeds could optionally be stirred in the solution using a magnetic stirrer or beads. EMS solution has a relatively short half-life of 48.5 h at 25°C. While the EMS solutions could be prepared 12–16 h before treatment, we recommend that they are freshly prepared to avoid inconsistency of the mutagen potency in replicates.
3. After 18 h of incubation, an equal volume of 10% sodium thiosulphate (w/v) solution was added to the EMS solution, gently stirred, and allowed to stand for 10 min. This aids in neutralizing the EMS solution and stopping the mutagenesis process. This step is important to ensure the reproducibility of results, especially when multiple seed batches are handled simultaneously.
4. Using long-handled tongs, the seeds bags were lifted from the neutralized EMS solution allowing excess liquid to drain off, and each seed bag was washed separately in 1000 ml water (in a 2000 ml beaker to avoid overflow) for a minimum of 10 min while occasionally stirring the solution gently to avoid splashes. The washing step was repeated five times.
5. Next, the EMS treated seeds were transferred to a wide pre-labeled weighing boat with blotting sheets and allowed to dry for at least 24 h.
6. Dried seeds were immediately planted into trays in the greenhouse or a growth chamber. Optionally the seeds could be stored at 4°C for a short duration before planting.

#### Precautionary measures in handling EMS

EMS is a powerful mutagen and extremely hazardous, therefore, special care (as described below) must be taken while handling the reagent or any contaminated material.

1. The personnel handling EMS must wear double layers of gloves with a long breakthrough time to prevent contact with the mutagen. Personnel should also wear appropriate personal protective equipment (PPE) listed in the materials section.
2. All materials contaminated with EMS should be collected and categorized separately as washable or disposable. Appropriate collection bags or buckets should be prepared before starting the experiment and EMS contaminated materials should be placed in designated buckets immediately after use. We recommend the use of three separate containers: for EMS contaminated glassware (washable), contaminated plastic ware and PPE (disposable solid waste), EMS liquid waste.
3. The contaminated glassware should be soaked in 10% sodium thiosulphate (w/v) solution for 12–15 h, followed by washing with water (using wash bottles) to neutralize and remove EMS. This soaking and washing solution should be discarded in the liquid EMS waste container. The glassware can then be cleaned with other labware.
4. The liquid EMS waste should be neutralized using 10% sodium thiosulphate (w/v) solution (1:1 dilution) and transferred to EMS waste container.
5. The efficiency of EMS mutagenesis is influenced by the temperature of the room and prepared solutions, and also the presence of catalytic ions (Zn^2+^, Cu^2+^). Therefore, the room/fume hood and the solutions to be used should be maintained at 25°C and deionized water should be used to ensure reproducibility of the procedure.
6. Magnetic stirrers or beads could be used to stir the EMS solution during overnight incubation of seeds. However, care must be taken to maintain low stirring speeds to avoid droplet dispersion.
7. It is advised to close the fume hood sash during the overnight incubation or when the fume hood is not actively monitored by the personnel. If the fume hood is in a shared lab, cautionary signage is highly recommended to avoid accidental contaminations.
8. It is essential to completely dry the seeds after EMS treatment, failure to do so could lead to degradation of seeds. Therefore, the use of large weighing boats to allow uniform dispersion and aeration of the seeds is recommended.
9. While handling EMS-mutagenized seeds, personnel should wear appropriate PPE and gloves. The growth chamber used for EMS-mutagenized seeds should be clearly labeled with cautionary signs.

### Physical mutagenesis

For field pea seeds, we chose 150, 200, 250, 300, 350, and 400 Gy doses with Cobalt-60 as the source for gamma radiations.

1. Approximately 200 seeds were aliquoted in nylon mesh bags for each dose and one for the control sample.
2. Seed bags were incubated in a vacuum desiccator over either silica, zeolite desiccant beads, or glycerol (60% v/v in water) for 1 week to equilibrate moisture content in all seeds.
3. Seed bags were then exposed to the desired gamma radiation dose using Gammacell 220 (SN:236) at the Saskatchewan Structural Sciences Centre, University of Saskatchewan, Saskatoon, Canada. This step was performed by a trained operator at the facility.
4. The radiation dose depends on the time of exposure to the radiation source and the dose rate. The exposure time is calculated as:

$$Exposuretime=\frac{Dose}{Doserate}$$

Gamma-irradiated seeds were immediately planted into trays in the greenhouse or a growth chamber or stored at 4°C for a short duration before planting.

#### Precautionary measures for gamma irradiation

1. The efficiency and reproducibility of physical mutagenesis are highly dependent on the moisture content of the starting material. Therefore, the steps involving equilibration of moisture content in seeds should not be skipped.
2. The efficiency and reproducibility of physical mutagenesis also depends on foreign material, such as dust or fiber. Therefore, care should be taken to minimize contamination.
3. Mutagenesis should not be performed on seeds infected with pathogens.

### Assessment of seed viability after EMS treatment and gamma irradiation

The assessment of seed viability to determine optimal doses of EMS and gamma radiation for pea mutagenesis was carried out based on the germination rate of treated seeds, which could be assessed by allowing the seeds to germinate on water-soaked Whatman sheets in a Petri dish. Although this is the most convenient method, it is also prone to microbial contaminations under prolonged incubation periods often caused by delayed germination of mutagenized seeds. Hence in our analysis, we analyzed the optimal dose on soil-grown plants as it allowed us to phenotype several post-germination traits, such as growth rate (time taken for the appearance of two-and four-leaves), and chlorosis. The control (non-mutagenized seeds) were also seeded at the same time for comparative analysis of phenotypes. To assess the effect of different EMS concentration and gamma radiation doses on seed viability and survival of pea seedlings, the germination of seeds, greening of cotyledon, and the appearance of second and fourth leaf was recorded for each of the treatment conditions.

### Gene editing in pea protoplasts

#### Mesophyll protoplasts isolation and purification

1. Pea seeds were surface sterilized with 75% ethanol for 1 min in a 15 ml centrifuge tube. After removing the ethanol using pipette, 10 ml of bleach solution was added and the tube was placed on a shaker for 15 min.
2. After 15 min, seeds were washed with sterilized water for five times and placed on germination media in a tissue culture cabinet.
3. After 3 weeks of subculturing leaf tissues were surface sterilized with 70% ethanol and then, using a new scalpel blade, cut into 0.5–1 mm pieces.
4. The pieces were quickly transferred to a plate containing 10 ml of 0.6 M mannitol and placed on a shaker for 10 min at 50 rpm in the dark.
5. The mannitol solution was removed and 10 ml of warm enzyme solution (0.6 M Mannitol, 10 mm MES, 1.5% Cellulase R-10, 0.75% Macerozyme R-10, 10 mm CaCl_2_, 0.1% BSA) was added. The plate went back onto the shaker at 50 rpm for 4 h in the dark.
6. Protoplasts were then transferred through a 40 μm filter, washed with W5 and layered overtop of 5 ml of 0.55 M sucrose in a round bottom culture tube.
7. Protoplasts were centrifuged for 10 min at 100 g in a swinging bucket without using brakes on at 4°C. The green intermediate layer was transferred to a sterile round bottom culture tube then 7 ml of W5 (5mm KCl, 154 mm NaCl, 125 mm CaCl2, and 2 mm MES pH 5.7) was added carefully.
8. After another centrifugation for 5 min at 100 g with brakes on maximum supernatant was discarded.
9. Afterwards 7 ml of W5 solution was added to purified protoplasts and was centrifuged for 2 min at 100 g with maximum brakes on.
10. Finally, supernatant was discarded and 1 ml of W5 solution was added to the purified protoplasts before estimating protoplasts yield using a haemocytometer.

#### PEG mediated protoplasts transformation

1. The purified protoplasts were re-suspended in appropriate amount of MMG solution with a desired concentration of 2 × 10^6^ protoplasts ml^−1^.
2. Thirty μg plasmid DNA was added to a 2 ml Eppendorf tube. Afterwards 200 μl of the protoplast solution was added to the tube and mixed gently.
3. An equal volume (230 μl) of the PEG solution was then added and mixed thoroughly by gently tapping and inverting the tube several times.
4. The DNA-protoplast-PEG mixture was incubated in the dark for 30 min in room temperature. After 30 min about 2x volume (900 μl) of W5 solution was added to the incubated mixture and mixed well by inverting the tube 2–3 times to stop the transformation process.
5. The mixture was then centrifuged for 5 min at 100 rpm.

#### Mutation detection in gene edited pooled protoplasts

1. CRISPR/Cas-mediated targeted mutations were detected by PCR using genomic DNA extracted from transfected pooled protoplasts after 72 h of transformation. The primers used in this study are listed in Supplementary Table 1.
2. The targeted gene was then amplified using gene-specific primers and the products were purified by 2% agarose gel electrophoresis in TAE buffer followed by extraction from excised gel bands using an appropriate kit (Qiagen, Hilden, Germany).
3. The purified products were digested with restriction enzymes with recognition sites incorporated in the gRNA mutation target, such that indels would destroy the site and prevent digestion.
4. PCR products that passed this rapid test for editing were then cloned in the TOPO vector system and were sequenced to confirm the mutation.
5. Sequence chromatograph were verified for multiple peaks near 3 bp upstream of the protospacer adjacent motif (PAM) region compared to the non-transformed control.

## Results

This study was aimed at establishing optimized protocols for chemical and physical mutagenesis of field pea. The pea seeds were exposed to varying doses of EMS and gamma radiation. Mutagenesis affects various stages of growth and development, especially in the M_1_ generation. Along with the deleterious effects of the mutagen on genome integrity, the non-genetic seed components, such as cellular constituents and enzymes are also affected (Talebi et al., 2012; Kumar et al., 2013; Serrat et al., 2014; Arisha et al., 2015). Here, we quantified several phenotypes at different growth stages of field pea to understand the biological effects of the mutagen and define an optimal dose for mutagenesis.

### Assessment of growth phenotypes following chemical mutagenesis

**Figure 1:**
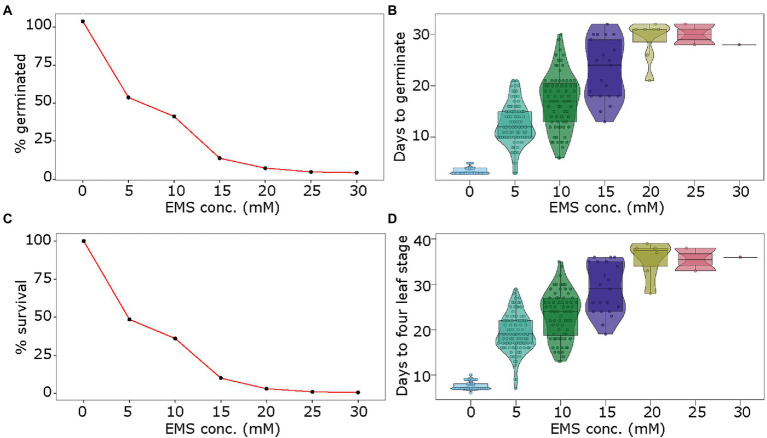
Optimization of EMS treatment for mutagenesis of pea seeds. Seeds of pea genotype CDC Amarillo were treated with different concentrations of EMS and were grown in the growth chamber. The germination rate **(A)**, days to germination **(B)**, survival rate **(C)**, and days to fourth-leaf stage **(D)** were assessed in treated and control samples to identify an optimal dose of EMS for mutagenesis of pea seeds.

The pea seeds were exposed to EMS doses of 5, 10, 15, 20, 25, and 30 mm. The seed germination rate dropped steadily from ~50% in 5 mm EMS to 37.5 and 10% in 10 mm and 15 mm EMS doses, and below 5% in the higher concentrations from 20 to 30 mm EMS (Figure 1A). Following a similar pattern, the delay in germination steadily increased with increasing EMS doses. While control treatment (0 mm) showed germination on average in 3 days, the EMS doses of 5, 10, and 15 mm showed germination at 12 days, 17 days, and 23 days, on average, respectively (Figure 1B). The higher doses of EMS (20, 25, and 30 mm) delayed germination to approximately 30 days (Figure 1B). The number of seedlings that survived post-germination followed an inverse pattern to increasing EMS doses. As opposed to 100% survival in control samples, the seeds treated with EMS doses of 5, 10, 15 mm showed 48.5, 36, and 10% seedling survival, respectively, while the higher doses (20, 25, and 30 mm) showed less than 5% seedling survival (Figure 1C). Among the surviving seedlings, we phenotyped the proportion of seedlings with aberrations in the chlorophyll accumulation, referred to here as chlorotic seedlings. The chlorosis in seedlings ranged from non-green patches of varying sizes on leaves to completely albino seedlings. These seedlings likely represent the germplasm with varying degrees of damage to chloroplast function-associated genetic material from chloroplast and nuclear genome. In our study, we observed 3 and 1.5% chlorotic seedlings in 5 and 10 mm EMS treatments, respectively, as compared to no chlorosis in control seedlings. As a measure of growth delay in mutagenized seedlings, we assessed the number of days required to reach the fourth-leaf stage. The control seedlings reached the fourth-leaf stage in approximately 1 week, while in seedlings treated with 5, 10, and 15 mm EMS the emergence of fourth-leaf occurred in approximately 19, 23, and 29 days, respectively (Figure 1D).

### Assessment of growth phenotypes following physical mutagenesis

**Figure 2:**
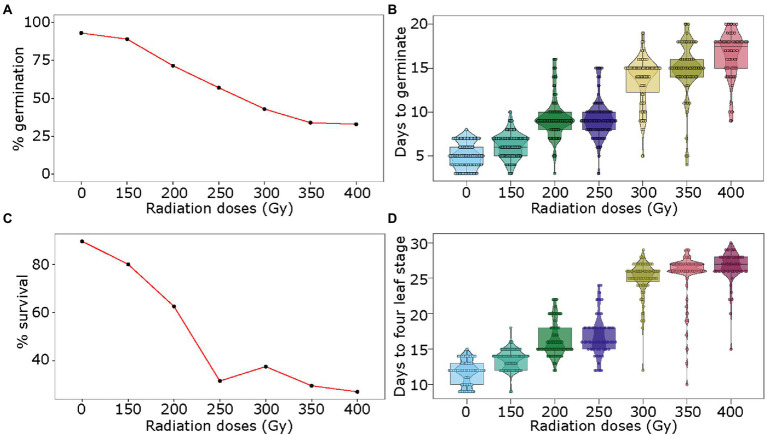
Optimization of gamma radiation treatment for mutagenesis of pea seeds. Seeds of pea genotype CDC Amarillo were treated with different doses of gamma radiation and were grown in the growth chamber. The germination rate **(A)**, days to germination **(B)**, survival rate **(C)**, and days to fourth-leaf stage **(D)** were assessed in treated and control samples to identify an optimal dose of gamma radiation for mutagenesis of pea seed.

For physical mutagenesis, the pea seeds were exposed to six doses of gamma radiation: 150, 200, 250, 300, 350, and 400 Gy. The germination rate was only marginally affected at 150 Gy at 89% as compared to 93% for the control sample (no radiation treatment; Figure 2A). Radiation treatment of seeds with 200 to 350 Gy showed steady reduction in germination from 71 to 34%, respectively (Figure 2A). The 400 Gy dose showed a similar effect on germination rate as the 350 Gy dose. Gamma irradiation also affected the number of days for germination. While the control seeds germinated on average at 5 days, the seeds irradiated with the lowest dose of 150 Gy germinated on average at 6 days (Figure 2B). The seeds treated with 200 and 250 Gy doses germinated on average at 9 days, delaying the germination by 4 days as compared to the control. Similarly, the doses of 300 and 350 Gy caused the seeds to germinate at 13 and 14 days, and the seeds treated with the strongest dose of 400 Gy showed germination at 16 days on average (Figure 2B). Further, the germinated seedlings derived from seeds irradiated with 150 and 200 Gy showed 80 and 62.5% survival rates, respectively (Figure 2C). However, the survival rate dropped between 32 and 27% of the germinated seedlings when the seeds were treated with gamma radiation doses of 250 to 400 Gy (Figure 2C). Of the surviving seedlings, as expected, the strongest radiation dose of 400 Gy showed the highest proportion of chlorotic plants (~ 14%), whereas lower doses from 150 to 350 Gy showed 2.5–7.5% chlorotic plants. Similar to chemical treatment, early growth retardation was observed for all gamma radiation doses. While the control sample reached the fourth-leaf stage in 12 days after germination, the seedlings from seeds treated with 150, 200, 250, 300, 350, and 400 Gy reached the fourth-leaf stage in 14, 16, 17, 25, 25 and 26 days, respectively (Figure 2D).

### Assessment of optimal doses for physical and chemical mutagenesis

**Figure 3:**
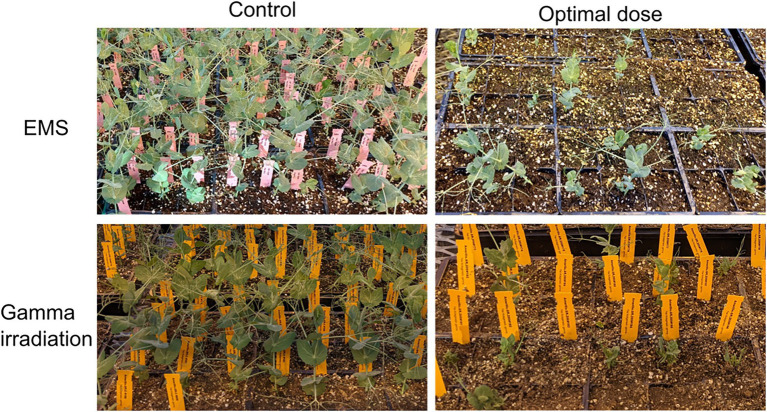
Comparative growth phenotypes of plants derived from pea seeds treated with optimized doses of mutagen and their respective controls. The upper panel shows growth phenotype of control (0 mm EMS, upper left panel) and optimal EMS dose (5 mm, upper right panel) treated M_0_ pea plants. The lower panel shows growth phenotype of control (untreated, lower left panel) and optimal gamma irradiation dose (250 Gy, lower right panel) treated M_0_ pea plants.

The optimal dose for mutagenesis is designated as the dose at which 50% lethality of seeds (LD_50_) occurs. For chemical mutagenesis of pea seeds, the lowest EMS concentration of 5 mm caused approximately 50% lethality and hence it was chosen as the optimal dose. At this optimal dose, 99.5% of the plants produced seeds. For physical mutagenesis, the radiation dose of 250 Gy caused approximately 50% lethality, however, only 30% of seedlings survived. On the other hand, the dose of 200 Gy showed 30% seed lethality, with 60% seedling survival (of the initial seeds) rate. Both of these doses showed comparable delays in germination and days to reach the four-leaves stage. Therefore, we selected an intermediate dose of 225 Gy as the optimal dose for physical mutagenesis in field pea. Figure 3 shows comparative phenotypes of pea plants treated with the optimal doses of EMS (upper right panel) and gamma-irradiation (lower right panel) as compared to their respective controls (left panels).

### CRISPR/Cas-based editing of LOX gene in pea protoplasts

**Figure 4:**
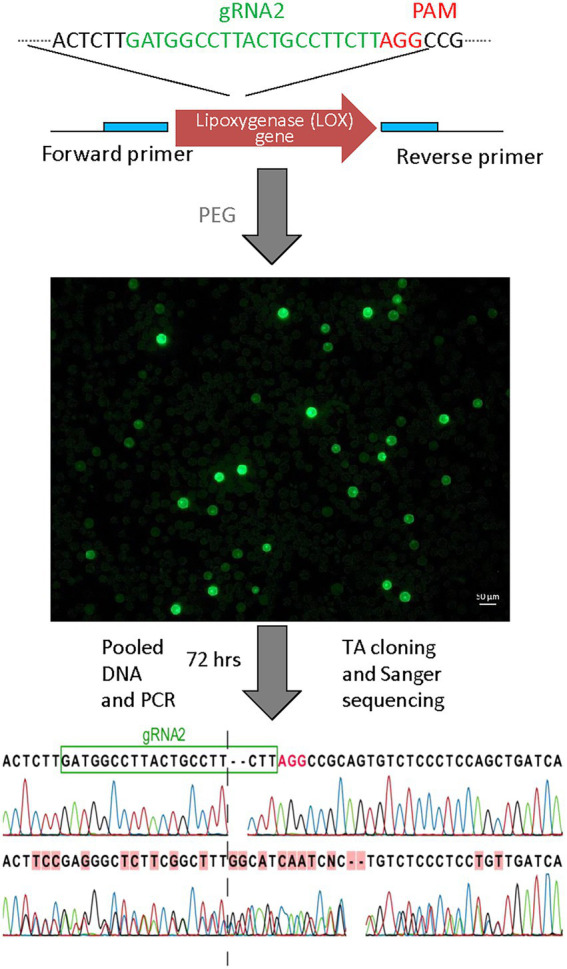
Targeted mutagenesis in pea protoplasts using CRISPR/Cas system. Sanger sequencing data showing evidence of mutation. LOX PCR product containing gRNA2 sites was sequenced. Dashed lines represent the DSB site of the gRNAs.

In this work we first established an efficient pea mesophyll protoplasts isolation and Poly Ethylene Glycol (PEG) mediated transformation system using green fluorescent protein (GFP) as reporter. We used the website^2^ to select the gRNA sequences from the first few exons and introns of the LOX gene. From the list generated, we chose the gRNA sequences with the highest CRISPRater scores which were located in exons and optimized the method for the first time as the evidence of successful CRISPR/Cas-mediated gene editing in pea protoplasts. The transformation efficiency (45% ±1.5) of pea mesophyll protoplasts using the described protocol was quantified by counting the percentage recovery of GFP reporter gene which was co-transfected and served as a control (Figure 4). The LOX PCR product from pooled DNA was gel purified and sequenced using Sanger sequencing (Figure 4). This study demonstrates the value and feasibility of gene editing in pea laying a technical foundation for future trait discovery and improvement of pea and other pulse crops.

## Discussion

Field pea is a leguminous break crop and an attractive option for sustainable agricultural practices (Tulbek et al., 2017). It is also a nutrient-rich food crop, and an alternate source of protein, starch, fiber, texturizer, and emulsifier for the exponentially growing plant-based industries (Tulbek et al., 2017). However, the use of field pea as a protein source requires reduction in anti-nutritional compounds, while the field pea-based processing industries require varieties with higher amounts of easily extractable protein, starch, and fiber to increase their profit margins. The establishment of field pea mutant and TILLING resources is an essential prerequisite for the rapid development of such varieties.

In this work, we developed optimized protocols for chemical and physical mutagenesis of pea. The procedures described here could be upscaled to generate a large-scale mutagenized population for pea improvement. We have identified 18 h of incubation in 5 mm EMS to be the optimal dose for chemical mutagenesis of field pea seeds. This concentration is lower than previous publications which optimized 20 mm EMS for the construction of the green pea TILLING platform and phenotyping database (Dalmais et al., 2008), or 0. 3% EMS (corresponding to ~24 mm EMS) for mutational breeding purposes (Sharma et al., 2009). In our study, 20–25 mm EMS treatment led to ~95% seed lethality, while Sharma et al. (2009), reported 53% lethality at 24 mm EMS treatment. Interestingly, Sharma et al. (2009), reported 1.93–3.45% chlorotic individuals in the M_1_ generation of 0.2–0.3% EMS (corresponding to 16–24 mm EMS) treated seeds, which is in the same range as 1.5–3% chlorotic individuals observed at 5–10 mm EMS treatment in our study. The variation in results could be attributed to pea varietal differences and the variable times of incubation in different studies. In this study, 18 h of incubation in EMS solutions were employed, while Dalmais et al. (2008) and Sharma et al. (2009) used 15 h and 8 h of incubation in EMS solutions, respectively. Additionally, Dalmais et al. (2008) and Sharma et al. (2009) used garden pea varieties, while we used a field pea cultivar in this study. Cultivar-specific differential responses to mutagens have also been reported previously (Çiftçi et al., 2006; Sharma et al., 2009), which highlights the importance of mutagen dose optimization for different plant cultivars. Interestingly, a previous report of gamma irradiation-mediated mutagenesis of green pea cultivars (Çiftçi et al., 2006) shows similar results as this study. Çiftçi et al. (2006) observed 63–80% and 73–90% germination in different green pea cultivars irradiated at 140 and 180 Gy, respectively, while we observed 89 and 71% germination at similar doses of 150 and 200 Gy, respectively.

Following the optimization of the mutagen doses, the decision of the number of seeds to be used for mutagenesis depends on the aim of the study and the available infrastructure. For building a mutant resource, a large number of seeds need to be mutagenized to ensure multiple mutations per gene or per unit genomic region. For example, the probability of identifying a mutation at a particular G:C base pair in the green pea TILLING database generated from ~4,700 M_1_ plants was calculated to be 0.06% (Dalmais et al., 2008). If this resource was generated from 50,000 mutant lines, the probability of identifying mutation at any G: C base pair would have increased to 52% (Dalmais et al., 2008).

Pea belongs to a recalcitrant group of plants in terms of regeneration. If the gene edited protoplasts can be regenerated into plants, protoplasts system could be used to target genes through transient transformation of CRISPR/Cas reagents into protoplasts. By selecting suitable target sites and achieving high editing efficiency, the target gene could be precisely mutated in all the cells of the regenerants if the gene editing event occurs before cell division. In such scenario, the mutation could be successfully transmitted to the offspring without chromosomal insertion of gene editing components. Nonetheless, the protoplast based gene editing system described here will be a valuable tool for functional genomics research in pea.

## Conclusion

We present step-by-step guide of conducting random and directed mutagenesis in commercially important legume crop, field peas. The optimized protocols for pea mutagenesis described here would be useful to create large-scale mutagenized populations to support gene discovery and crop improvement in field pea. The general principles and mutagenesis methods described here also provide a foundation for similar studies in other leguminous crops.

## Data availability statement

The original contributions presented in the study are included in the article/Supplementary material, further inquiries can be directed to the corresponding authors.

## Author contributions

SK conceived and designed the study. PP performed the chemical and physical mutagenesis experiments. PB performed the gene editing experiments. PP and PB prepared the first draft of the manuscript. SK edited and finalized the manuscript. All authors contributed to the article and approved the submitted version.

## Funding

This work was supported by the Aquatic and Crop Resource Development Centre as part of its contribution to the Sustainable Protein Production Program of National Research Council Canada.

## Conflict of interest

The authors declare that the research was conducted in the absence of any commercial or financial relationships that could be construed as a potential conflict of interest.

## Publisher’s note

All claims expressed in this article are solely those of the authors and do not necessarily represent those of their affiliated organizations, or those of the publisher, the editors and the reviewers. Any product that may be evaluated in this article, or claim that may be made by its manufacturer, is not guaranteed or endorsed by the publisher.

## Footnotes

^1^ https://mvd.iaea.org/

^2^ https://crispr.cos.uni-heidelberg.de
