# Supplementary material for: Optimized methods for random and targeted mutagenesis in field pea (Pisum sativum L.)
Source: Front Plant Sci. 2022 Sep 8;13:995542. doi: 10.3389/fpls.2022.995542 (PMC9498975; doi:10.3389/fpls.2022.995542)
Supplement: Supplementary file 1 [file Table_1.DOCX]

| **Supplementary table 1:** List of primers used in the study | |
| --- | --- |
| **Name** | **Primer Sequence** |
| Lipoxygenase Forward | 5’- ATGTTTCCAAATGTGACAGGACTCC-3’ |
| Lipoxygenase Reverse | 5’-CTTGTATCTAACCTTTCCTCGTTGG-3’ |
| Kanamycin Forward | 5’- TGAGAATTAAGGGAGTCACGTTATGACC-3’ |
| Kanamycin Reverse | 5’- CGGCCATTTTCCACCATGATATT-3’ |
| gRNA1 | 5’- CGGCCATTTTCCACCATGATATT - 3’ |
| gRNA2 | 5’- CTTGTATCTAACCTTTCCTCGTTGG -3’ |
